# Long-Term Intranasal Nerve Growth Factor Treatment Favors Neuron Formation in *de novo* Brain Tissue

**DOI:** 10.3389/fncel.2022.871532

**Published:** 2022-07-19

**Authors:** Nina Colitti, Franck Desmoulin, Alice Le Friec, Wafae Labriji, Lorenne Robert, Amandine Michaux, Fabrice Conchou, Carla Cirillo, Isabelle Loubinoux

**Affiliations:** ^1^Toulouse NeuroImaging Center (ToNIC), Inserm, University of Toulouse (UPS), Toulouse, France; ^2^Unit of Medical Imaging, National Veterinary School of Toulouse, University of Toulouse, Toulouse, France

**Keywords:** nerve growth factor, intranasal, acute brain lesion, regeneration, MRI

## Abstract

**Objective:**

To date, no safe and effective pharmacological treatment has been clinically validated for improving post-stroke neurogenesis. Growth factors are good candidates but low safety has limited their application in the clinic. An additional restraint is the delivery route. Intranasal delivery presents many advantages.

**Materials and Methods:**

A brain lesion was induced in twenty-four rats. Nerve growth factor (NGF) 5 μg/kg/day or vehicle was given intranasally from day 10 post-lesion for two periods of five weeks, separated by a two-week wash out period with no treatment. Lesion volume and atrophy were identified by magnetic resonance imaging (MRI). Anxiety and sensorimotor recovery were measured by behavior tests. Neurogenesis, angiogenesis and inflammation were evaluated by histology at 12 weeks.

**Results:**

Remarkable neurogenesis occurred and was visible at the second and third months after the insult. Tissue reconstruction was clearly detected by T2 weighted MRI at 8 and 12 weeks post-lesion and confirmed by histology. In the new tissue (8.1% of the lesion in the NGF group *vs.* 2.4%, in the control group at 12 weeks), NGF significantly increased the percentage of mature neurons (19% *vs.* 7%). Angiogenesis and inflammation were not different in the two groups. Sensorimotor recovery was neither improved nor hampered by NGF during the first period of treatment, but NGF treatment limited motor recovery in the second period.

**Interpretation:**

The first five-week period of treatment was very well tolerated. This study is the first presenting the effects of a long treatment with NGF and has shown an important tissue regeneration rate at 8 and 12 weeks post-injury. NGF may have increased neuronal differentiation and survival and favored neurogenesis and neuron survival through subventricular zone (SVZ) neurogenesis or reprogramming of reactive astrocytes. For the first time, we evidenced a MRI biomarker of neurogenesis and tissue reconstruction with T2 and diffusion weighted imaging.

## Highlights

- To date, no safe and effective pharmacological treatment has been clinically validated for improving post-stroke neurogenesis.
- NGF improved neuron differentiation and survival in a rat model of stroke.
- Safety of long-term treatment with growth factors needs careful investigation.
- Intranasal NGF delivery during 10 weeks is a possible delivery route.
- We demonstrate a novel MRI biomarker of neurogenesis.

## Introduction

Stroke is the leading cause of acquired severe disability in adults. Most strokes lead to lesions of the brain motor areas causing motor impairment. Motor recovery takes months and more than 50% of patients remain severely disabled on the upper limb (Lawrence et al., 2001; Kwakkel et al., 2003; Donnan et al., 2008).

Stroke causes cell death, destruction of the extracellular matrix, inflammation and secondary lesions, as well tissue repair (Cirillo et al., 2020) for example through the formation of the glial scar, a defense mechanism that prevents the lesion from spreading and aids axon regeneration (Burda and Sofroniew, 2014; Anderson et al., 2016). The brain has the amazing, though limited, ability to reconstruct itself by generating new neurons from stem cells. After a stroke, neurogenesis increases between the first and fourth week (Jin et al., 2001; Arvidsson et al., 2002). Nevertheless, the survival rate of new neurons is too low (0.2%) to recover from the injury (Arvidsson et al., 2002). This low survival is also due to the lack of growth factors, normally released by the macro- and microglia and stored within the extracellular matrix (Khelif et al., 2018).

Decreasing inflammation and promoting neurogenesis are pivotal to render the lesion environment favorable to tissue regeneration after stroke. Preclinical studies have shown that growth factors may have these abilities (Thorne et al., 2004; Grinberg et al., 2017). The current study focuses on Nerve Growth Factor (NGF), a key molecule with neuroprotective, anti-inflammatory, and stimulating neurogenesis effects (Thoenen and Barde, 1980; Zhu et al., 2011). Importantly, NGF has already been tested in patients with dementia (de Bellis et al., 2018) or Alzheimer’s disease (Jönhagen et al., 1998) and showed promising effects (Aloe et al., 2012). Nevertheless, the therapeutic use of growth factors remains a challenge, and finding the optimal dose is a compromise between efficacy and safety. For example, one trial testing basic Fibroblast Growth Factor in acute ischemic stroke patients showed serious adverse events, decreased blood pressure, and increased mortality, and was therefore prematurely interrupted (Bogousslavsky et al., 2002). In addition, NGF may cause hyperalgesia when injected intramuscularly (Costa et al., 2020). Furthermore, the dose of growth factors must be efficacious. However, no double-blinded randomized clinical trial on growth factors given in the subacute stroke phase has yet evidenced functional efficacy. Two large clinical trials in stroke patients treated with erythropoietin or colony-stimulating factor showed no functional improvement (Bath et al., 2013).

Pharmacological therapy in brain injury diseases like stroke is inherently challenging due to the blood-brain barrier (BBB). Neurotrophic factors, for example, are often too large to pass the BBB. The intranasal pathway appears attractive in this context. Through this route, a molecule arrives directly to the brain following the olfactory and trigeminal nerves pathways (Balin et al., 1986; Thorne and Frey, 2001), allowing a high bioavailability in the brain (Agrawal et al., 2018). Additionally, intranasal delivery is non-invasive and is well-tolerated by patients (Hanson and Frey, 2008).

This study merges the interest in NGF and the intranasal route in a preclinical model of brain lesion, validated in rats (Davoust et al., 2017; Cirillo et al., 2018). Tissue repair (glial scar thinning) and regeneration (endogenous neuro-, glio- and angiogenesis), as well functional recovery (sensorimotor behavior) were evaluated. Our primary goal was to assess NGF-mediated neurogenesis. Following injury, sensorimotor impairment and anxiety were assessed, and the lesion was characterized by anatomical Magnetic Resonance Imaging (MRI) and *post-mortem* histology. Recovery and tissue reconstruction are prolonged processes and the long-term treatment to achieve substantial efficacy must be safe. Thus, for the first time, our study also enabled to test the safety of prolonged treatment with NGF, as a secondary goal.

## Materials and Methods

### Animals

Female 10-weeks-old Sprague-Dawley rats (280–320 g, Janvier, France) were used in this study. Rats were housed two per cage (30 cm length × 18 cm height × 32 cm width), in a climate-regulated environment (20°C) under a 12 h/12 h light/dark cycle (lights on at 7:00 AM) with free access to food and water. Animals were treated according to the Council of the European Communities guidelines (EU Directive 2010/63). This protocol was approved by the “*Direction Départementale de la Protection des Populations de la Haute – Garonne*” and the “*Comité d’éthique pour l’expérimentation animale Midi-Pyrénées*” (protocol n° 16780). All efforts were made to minimize the number of animals used and the suffering they experienced. All the experiments have been reported following the ARRIVE guidelines.

### Surgical Procedures

The malonate model has the advantage of being able to target specific and key brain regions involved in human ischemic stroke, such as the sensorimotor cortex and the corticospinal tract (Vaysse et al., 2015a,b; Davoust et al., 2017) or the internal capsule (Cirillo et al., 2018). Surgery is quick and does not present any specific difficulty. The extent of the lesion can be easily controlled by the volume of toxin injected. Malonate creates a chemical energy failure by blocking the Krebs cycle and respiratory chain enzymes and thus is close to ischemia in that it induces an excitotoxic lesion with a penumbra (Greene and Greenamyre, 1996; Schulz et al., 1998; Millerot-Serrurot et al., 2007). Though this model does not completely mimick ischemic stroke because it does not cause vascular occlusion, it avoids vascular variability and therefore has the advantage of low variability in lesion size. The malonate model is thus suitable for comparisons of groups in pharmacological studies involving time-consuming experiments (behavior, MRI, histology).

**FIGURE 1:**
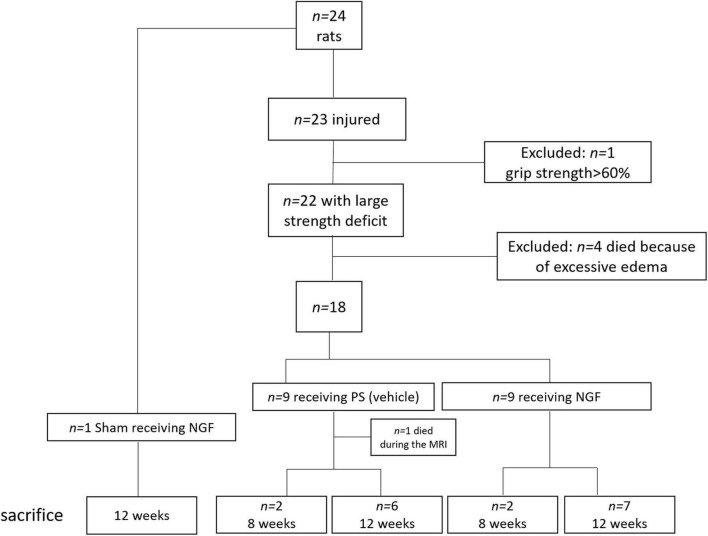
Flowchart of the study, with the details on the rats excluded from the protocol: *n* = 4 died after the surgery (excessive edema); *n* = 1 excluded because of light deficit and performance >60% at the grip strength test. Rats were sacrificed at 8 weeks (*n* = 2 per group) and 12 weeks (*n* = 7 per group) to evaluate the presence of reconstructed tissue at the different time points. PS: physiological saline; NGF: nerve growth factor; MRI: Magnetic Resonance Imaging.

Rats were anesthetized with isoflurane (3% for induction, 3–5% for maintenance, in 0.7 l/min O_2_), secured to a stereotaxic frame (Bioseb lab, France). Pre-medication with an intraperitoneal injection of methylprednisolone (20 mg/kg, Centravet, France) and a subcutaneous injection before the scalp incision of lidocaine 2% (4 mg/kg, Centravet) were used for analgesia. Body temperature, measured by a rectal probe, was maintained at 37°C using a homeothermic blanket. Cortical lesion of the motor area (M1) was induced by malonate injection [5 μL, 3M solution, pH 7.4 in phosphate buffer saline (PBS); Sigma-Aldrich, France] in *n* = 23 rats at the following stereotaxic coordinates: 2.5 mm lateral and 0.5 mm ahead to Bregma, with 2 mm depth (Paxinos and Watson, 2007). PBS was used for the sham (*n* = 1). The lesioned hemisphere was the dominant one, identified thanks to the grip strength test (see below). This model was previously validated and published by our group (Vaysse et al., 2015a; Davoust et al., 2017). One day after the surgery, *n* = 4 rats died because of excessive edema. Only animals that displayed substantial neurological deficits 1 week after injury (grip strength < 60% of pre-lesion value) were selected for the protocol (*n* = 1 excluded rat) (flowchart in Figure 1). Animals were matched according to the extent of the lesion size evaluated by MRI, the grip and neurological scale performance, and assigned to the different groups (vehicle *vs*. NGF).

### *In vivo* Magnetic Resonance Imaging

*In vivo* MRI was performed with a 7T Biospec animal imager (Bruker Biospin, Ettlingen, Germany). Rats were scanned at three time points: pre-injury, 24-h post-injury and at 8 (*n* = 4) or 12 weeks (*n* = 16) post-injury. Before the imaging, rats were anesthetized with isoflurane (3% for induction, 1% for maintenance, in 0.7 l/min O_2_). In order to identify the edematous lesion, T2 weighted images using a TurboRare sequence were acquired: TR = 6830.5 ms, TE = 35.8 ms, NSA = 9, FOV = 48 × 48 mm^2^, matrix 320 * 320, 60 slices, resolution 150 × 150 × 300 μm, acquisition time 41 min. Spin echo weighted diffusion images were also acquired: TR = 1000 ms, TE = 25 ms, FA = 90°, NSA = 3, FOV 33 × 33 mm^2^, matrix 82 * 82, 30 slices, resolution 400 × 400 × 800 μm, reconstructed slice thickness 600 μm, b value 650 s/mm^2^, 12 diffusion gradient directions, acquisition time 40 min.

### Behavior Tests

To evaluate sensorimotor functions, rats were trained two weeks before the surgery for the grip strength test and the neurological severity score assessment. Behavior tests were performed one week before the injury (baseline), then 48 h post-injury, every week during the first month and every month for the following two months. Each time point was evaluated in triplicate on three different days within the same week. Two additional tests were used in the study: the limb-use asymmetry test and the light-dark box test. These were performed three times: one week pre-injury, one-week post-injury and eight weeks post-injury. The experimenters were blind to the treatment throughout the study. For all tests, each value reported represents the median ± interquartile range [first quartile (Q1); third quartile (Q3)] of each group.

#### Grip Strength Test

The grip strength test measures the maximal forelimb muscle grip strength. The experimenter restricts the rat by holding it by the back and leaving the forelimbs free. Instinctively, the rat grabs the horizontal bar of the Grip device (Bioseb, France), and is gently pulled back until the grasp is broken. The connected dynamometer measures the maximum isometric force (in Newton). This test determines the dominant paw of the rat. This test was validated in previous studies by the group (Demain et al., 2015; Davoust et al., 2017; Cirillo et al., 2018). All rats underwent the strength test (3 trials/paw/day) on 3 consecutive days, and a mean score was calculated for each week, before and after the injury. Values of the contralateral forelimb were normalized by values of the ipsilateral forelimb.

#### Neurological Severity Scale

The NSS includes five tests to evaluate sensorimotor function (reflexes, stability), sensitivity (proprioception) and depression. Motor ability was tested with 1/suspension by the tail where a healthy rat stretches its limbs while an injured rat retracts them, and 2/by hanging the animal two centimeters above a bench to determine if the rat is able to touch the top and move forward on its paws (healthy reaction). The evaluation of proprioception consists in putting the lesioned forelimb and hind limb to the edge of a bench. Injured rats let the limbs hanging down whereas healthy ones instinctively and quickly put the limbs back on the bench. The Beam Balance Test was used to assess the balance, motor and sensitive abilities of the rat, which normally tries to secure itself on the beam using its four limbs. When the sensorimotor cortex is lesioned, the limbs hang down from the edge of the beam. Finally, grooming behavior was observed since a lack of grooming in rats can reflect “pseudo-depression” (Kalueff and Tuohimaa, 2005). All the tests were scored in a scale from 0 to 16 points. The higher is the score, the more severe are the deficits [adapted from Vaysse et al. (2015b); Cirillo et al. (2018)].

#### Limb-Use Asymmetry Test

The asymmetry test allows to evaluate motor deficits of the whole limb (shoulder, elbow, paw) over time. Forelimb use during explorative activity was analyzed in a clean cage (one rat at a time) by video recording for 3 min. This test assesses spontaneous forelimb use. Normally, rats prefer symmetrical vertical exploration (rearing up and supporting themselves using both forelimbs). After injury, the preference for using the unimpaired forelimb (asymmetry) is evaluated by counting the number of times the rat uses its ipsilateral or contralateral paw against the wall of the cage (Schallert et al., 2000). The percentage of forelimb use is calculated as follows: Asymmetry (%) = number of times the ipsilateral paw is used for support - number of supports for the contralateral paw/total number of supports * 100.

#### Anxiety Test

The light-dark box test evaluates anxiety based on the aversion to bright light and spontaneous exploratory behavior in a new environment. A box is divided into two equal areas, with a dark area and a sliding door system to access an illuminated area. Rats are placed into the dark side, door closed, for 30 s, then the experimenter opens the door to a quarter. The latency to exit the dark side and enter the light compartment is recorded (Vahid-Ansari et al., 2016). The test stops when the rat is completely out with its four paws, with a time limit of 5 min.

### Intranasal Administration of Nerve Growth Factor

**FIGURE 2:**
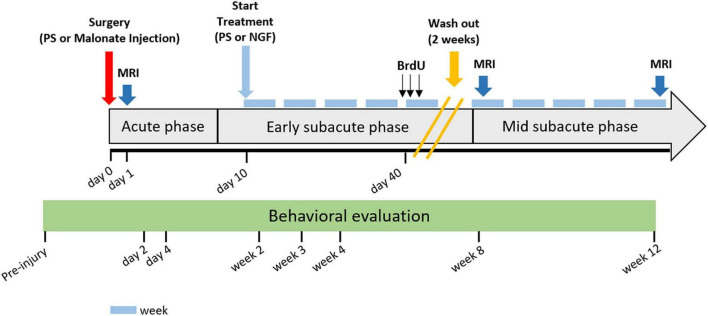
Detailed study protocol. Behavioral tests started before injury and were performed at time points: day 2 and 4, and week 2, 3, 4, 8, and 12 post-injury. Intranasal administration of PS (vehicle) or NGF started at day 10 post-injury and lasted for 10 weeks (5 weeks + 5 weeks with 2 weeks of wash-out). MRI acquisitions were performed at day 1- and 8-weeks or 12-weeks post-injury. The gray arrow indicates stroke evolution: acute phase until day 7, early subacute phase from day 7 to week 8 post-injury; mid subacute phase from week 8 to 12 post-injury. Each light blue-dash indicates one week. Yellow oblique-parallel lines on the gray arrow indicate the wash-out period. PS: physiological saline; NGF: nerve growth factor; MRI: magnetic resonance imaging; BrdU: 5′-bromo-2-deoxyuridine.

Animals were anesthetized with isoflurane (3% for induction) and placed in a supine position. NGF (Alomone labs, Israel) was dissolved in 0.9% physiological saline and delivered intranasally at a dose of 5 μg/kg/d (Lv et al., 2013), in a volume of 10 μl per nostril *via* a flexible catheter inserted 1 cm inside the nostril, connected to a 25 μl Hamilton syringe. Rats in the vehicle group received 0.9% saline with the same procedure. Treatment started 10 days after surgery, and was given 5 times a week for two periods of 5 weeks spaced two weeks (Figure 2). The two-week wash-out provided an opportunity to check for potential side effects of daily isoflurane anesthesia. If daily anesthesia impacts recovery, we would expect to observe slow recovery in the first period (because of anesthesia toxic effect), followed by an increase in recovery during the wash out period and then a slowing when anesthesia was resumed.

### Injection of 5-Bromo-2-Deoxyuridine

To count proliferating cells and assess late neurogenesis and cell survival, rats received intraperitoneal injections of 5-bromo-2-deoxyuridine (BrdU; 50 mg/kg in 0.9% NaCl; Sigma-Aldrich two times a day with 9 h intervals, for 3 consecutive days). The injections were performed 40 days after the lesion. Since it was shown that most neo-neurons died within 5 weeks (Arvidsson et al., 2002), we wanted to evaluate whether late neurogenesis occurs and is modified by NGF treatment. Four rats were sacrificed 2 weeks after the end of BrdU injections (8 weeks post-lesion) and the remaining 16 rats 6 weeks later (12 weeks post-lesion).

### Plasma Enzyme-Linked Immunosorbent Assay

To test whether chronic NGF treatment had an impact on growth factor production, we measured plasma levels of Brain-derived neurotrophic factor (BDNF) and Vascular endothelial growth factor (VEGF). Blood was taken by intracardiac puncture after euthanasia, collected in tubes with Ethylene diamine tetraacetic acid (EDTA) (*n* = 11: 1 sham, 8 vehicle and 9 NGF) and centrifuged at 2500 rpm for 15 min at 4°C to obtain the plasma. VEGF and BDNF assays were carried out with the Quantikine Enzyme-linked immunosorbent assay kits (#RRVOO and #DPNT00 R&D Systems/Biotechne, France). Samples were measured using a microplate reader at a wavelength of 450 nm.

### Tissue Histology

At the end of the study, rats (*n* = 19) were anesthetized with isoflurane and sacrificed with a lethal intraperitoneal injection of pentobarbital (160 mg/kg, Centravet). Intracardiac perfusion of heparinized 0.9% NaCl (200 ml, 20 min) eliminated blood from the vessels, and was followed by 4% paraformaldehyde (PFA) (250 to 300 ml, 40 min) for fixation. The brain was then extracted and immersed in a 4% PFA bath for 24 h of post-fixation at 4°C and washed in two successive baths of PBS. Sucrose baths of increasing concentration (10, 20, and 30%) were used for cryoprotection. Approximately 500 20 μm coronal brain sections were cut with a microtome (Sliding Microtome Microm HM 450, Thermo Scientific, Germany). One in every twelve slices was stained with Cresyl Violet acetate, according to standard procedure (Nissl staining). Cresyl violet stained slides were scanned on a 3DHISTECH’s Slide Converter, and these images used for the quantification of reconstructed tissue area. The Slide software drawing tool allows to define the reconstructed tissue area, for each brain section. This tissue is loose and therefore appears lighter and the cells are spatially disorganized. We calculated the total area of reconstructed tissue in μm^2^ multiplied by the antero-posterior distance in μm from the first to the last section where reconstructed tissue was observed. These two factors were divided by the MRI lesion volume in mm^3^ at 12 weeks to obtain the percent (%) of reconstructed tissue normalized to the size of the lesion, as follows (Table 1): Reconstructed Tissue % = $\frac{N\,e\,o\,t\,i\,s\,s\,u\,e\,A\,r\,e\,a*A\,P\,D\,i\,s\,t\,a\,n\,c\,e}{L\,e\,s\,i\,o\,n\,v\,o\,l\,u\,m\,e}$.

**TABLE 1:** Volumes of reconstructed tissue and magnetic resonance imaging (MRI) lesion volumes measured 12 weeks post-injury.

Group	*n*	Total area of reconstructed tissue Median (mm^2^) IQR	Antero-posterior distance Median (mm) IQR	Lesion volume Median (mm^3^) IQR	RT (% IQR)
Vehicle	6	2.71± 4.35	2.16 ± 2.91	208 ± 69	2.42 ± 7.14
NGF	7	4.17± 4.00	1.80 ± 1.08	173 ±14	8.19 ± 14.13

*IQR: interquartile range; RT: reconstructed tissue; NGF: nerve growth factor.*

**FIGURE 3:**
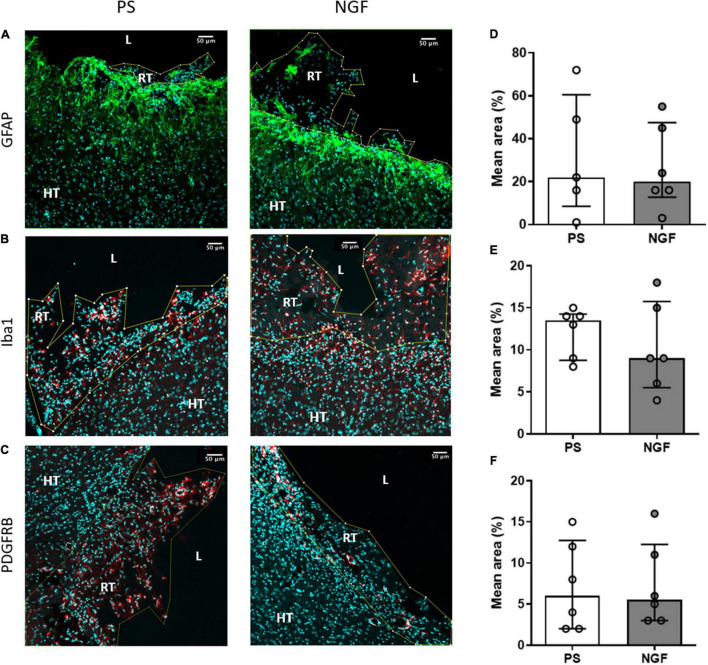
Histology of brain sections and characterization of the reconstructed tissue and edge of the lesion: glio- and angiogensesis. Representative images and quantification of GFAP **(A,D)**, Iba1 **(B,E)** and PDGFRB **(C,F)** mean intensity in PS (*left*) and NGF (*middle*) groups. There was no significant difference between the groups, as reported in the graphs **(D–F)**. All the graphs show the distribution of individual values and the median and the interquartile range. PS group: *n* = 6; NGF group: *n* = 7 Scale bars: 50 μm. PS: physiological saline; NGF: nerve growth factor; GFAP: glial fibrillary acidic protein; PDGFRB: platelet-derived growth factor receptor beta; L: lesion; RT: reconstructed tissue; HT: healthy tissue.

The other brain sections were used for immunofluorescence staining. Floating sections were incubated in PBS buffer 0.1% Triton X-100 (Sigma-Aldrich) and 4% serum (donkey or goat, Thermo Fisher Scientific or Sigma-Aldrich, respectively), for 2 h at room temperature. Sections were exposed to the primary antibodies, overnight at 4°C. After three washes with PBS, sections were incubated with a specific secondary antibody coupled to a fluorochrome, for 2 h at room temperature (antibody list in Table 2). 4′,6-diamidino-2-phenylindole (DAPI) (1/4000, Sigma-Aldrich) was used to counterstain the nuclei and sections were mounted using the Dako fluorescent mounting medium (Agilent, France). Images were captured using a confocal fluorescence microscope Zeiss LSM710 (Cell Imaging facility, Toulouse Institute for Infectious and Inflammatory Diseases) and analyzed using the ImageJ and Fiji image analysis software (Schindelin et al., 2012). To measure glial scar thickness, 16-bit images of Glial fibrillary acid protein (GFAP)-stained sections were analyzed (*n* = 3 images per section, Z stack acquisition with confocal microscope, *n* = 6 for PS group and *n* = 7 for NGF group). The GFAP-positive glial scar was manually thresholded by an experimenter blinded to the experimental group. The thresholded glial scar crosses the image, so that after binarization, we obtain two “objects” (healthy tissue) separated by the scar. The distance between the two objects corresponds here to the distance between the two outer borders of the scar (where the scar meets the healthy tissue). This distance was computed using Fiji’s voronoi function, the output of which gives the half thickness of the glial scar, that is finally multiplied by two. To evaluate neurogenesis, quantification was made only in the identified reconstructed tissue where the lesion took place before. This reconstructed tissue is identifiable beyond the GFAP+ glial scar (see Figure 3A). Based on the Cresyl violet staining, one adjacent section per rat was chosen because it represents the slice with the most reconstructed tissue. Three counting frames (708 x 708 μm^2^) per section are quantified. Surface quantification was used for cytoplasmic markers: Doublecortin (DCX) and beta 3 tubulin [three counting frames (708 x 708 μm^2^) per section, 20 × magnification, Z stack acquisition with confocal microscope; *n* = 6 for PS group and *n* = 7 for NGF group]. Mature neurons were quantified by counting their Neuron specific nuclear protein (NeuN)-positive nuclei in the identified reconstructed tissue (three counting frames per section, expressed as percent of DAPI+ cells, 20 × magnification, Z stack acquisition with confocal microscope; *n* = 6 for PS group and *n* = 7 for NGF group). Finally, BrdU+ cells were quantified in the same way (*n* = 4 for PS group and *n* = 4 for NGF group). For Iba1 and Platelet-derived growth factor receptor beta (PDGFRB) markers, the same surface quantification procedure was applied. Quantification of the PDGFR staining was based on the detection of vessel-resembling structures by thresholding the highest signal intensities.

**TABLE 2:** List of primary and secondary antibodies used in the study.

Primary antibody (catalog #)	Host species	Dilution	Secondary antibody
GFAP (Dako #Z0334)	rabbit	1/1500	Goat anti-rabbit 488 Alexa Fluor
Iba1 (Abcam #ab5076)	goat	1/500	Donkey anti-goat 568 Alexa Fluor
DCX (SantaCruz #sc-8066)	goat	1/250	Donkey anti-goat 568 Alexa Fluor
NeuN (Abcam #ab177487)	rabbit	1/300	Donkey anti-rabbit 594 Alexa Fluor
beta 3 tubulin (Covance #PRB435P)	rabbit	1/500	Donkey anti-rabbit 594 Alexa Fluor
PDGFRB (Abcam #ab32570)	rabbit	1/100	Donkey anti-rabbit 594 Alexa Fluor

*GFAP: glial fibrillary acidic protein; Iba1: ionized calcium-binding adapter molecule 1; DCX: doublecortin; NeuN: neuron specific nuclear protein; PDGFRB: Platelet Derived Growth Factor Receptor Beta.*

### Statistical Analysis

Comparisons between two groups were made using the Mann-Whitney U-test. The Kruskal-Wallis test was used for comparisons of more than two groups. Post-hoc comparisons between groups were made using the Mann-Whitney U-test. Bonferroni correction was used to correct for multiple testing. The effect of the lesion was assessed between pre-lesion and post-lesion times with a Wilcoxon test. Functional recovery was evaluated between post-lesion and end-point times with a Wilcoxon test. Spearman’s test was used to identify correlations. Results were presented as median (first quartile Q1; third quartile Q3). GraphPad Prism software was used for the statistical analysis.

## Results

### MRI Advantages for Pharmacological Assessment

#### *In-vivo* Magnetic Resonance Imaging

**FIGURE 4:**
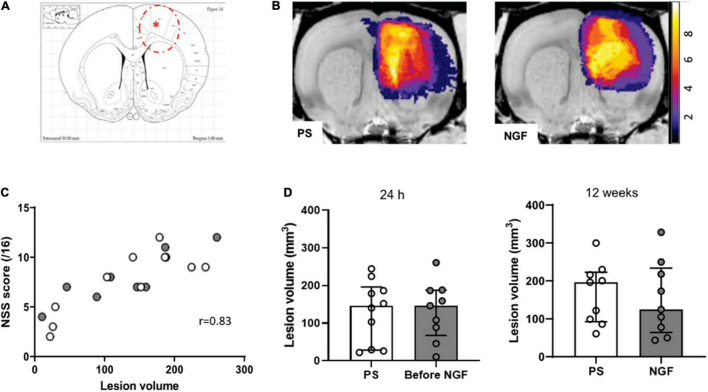
Characterization of the lesion. **(A)** Coronal section corresponding to the malonate injection site (red asterisk) and lesion size (red dashed line), adapted from Paxinos Atlas. **(B)** Representative T2-weighted MRI color-coded images showing the localization of the lesion in the two groups of rats (PS: *left panel*; NGF: *right panel*). The panels show the overlap map of injured voxels, providing an overview of the lesioned brain areas 24 h after the injection of malonate for the groups. Color indicates the number of rats injured at each voxel. **(C)** Graph showing the correlation between the lesion volume 24 h after the injection of malonate and motor deficits (NSS score) measured 48 h post-injury (*p* < 0.0001). **(D)** Notch box plots showing the distribution of lesion volumes, expressed in mm^3^, 24 h (*left*) and 12 weeks (*right*) post-injury. PS group: *n* = 9; NGF group: *n* = 9. PS: physiological saline; NGF: nerve growth factor; NSS: neurological severity scale; MRI: magnetic resonance imaging.

Twenty-four hours after the injury (malonate injection in the M1 region, Figure 4A), T2-weighted MRI made it possible to characterize lesion volume variability. Rats assigned to NGF and PS treatment groups were matched for lesion size before the start of treatment so that there was no difference between groups for the lesion volume at 24 h post-injury (Figure 4D). Thus, MRI volume assessment can then solve the problem of lesion volume variability in animals before the initiation of a treatment. The analysis of the evolution of the lesion volumes 24 h and 12 weeks post-injury showed that the sensorimotor cortex was largely affected (Figure 4B) in both groups. Interestingly, a correlation was found significant between the 24 h lesion volumes and the sensorimotor deficits 2 days post-injury (Figure 4C). For future studies, this correlation represents a valid and useful way to indirectly assess lesion volumes via our neurological sensorimotor scale when 24-h post-injury MRI information would be unavailable. Then, matching paired animals would be easily possible between groups.

We found that the treatment with NGF did not influence the T2 hyperintense MRI lesion volumes (Figure 4D). Before the treatment, the median lesion volume at 24 h was 146.5 mm^3^ (Q1 = 67.4; Q3 = 187.5 mm^3^) for the NGF group and 146.3 mm^3^ (Q1 = 47.9; Q3 = 184.5 mm^3^) for the PS group (Figure 4D, *left*). At the end of the study (12 weeks post-injury), the atrophy and hypertrophied ventricle was included in the lesion volume. The median lesion volume was 124.6 mm^3^ (Q1 = 43.4; Q3 = 328.1 mm^3^) for the NGF group and 196.5 mm^3^ (Q1 = 60.6; Q3 = 299.9 mm^3^) for the PS group (Figure 4D, *right*). The NGF group had similar lesion volume compared to the PS group 12 weeks post-injury (*p* = 0.73). Cytotoxic edema, hyperintense on diffusion-weighted images at 24 h, reveals dead tissue (Figures 5.1A,B). In the very same areas, vasogenic edema is detected by T2 imaging. As described in the literature (Zille et al., 2012), the T2 value increases as the content in free water increases and the tissue becomes more necrotic so that the lesion is T2 hyperintense. Accordingly, T2 signal intensity was significantly higher in the lesion compared to the contralateral normal tissue, + 85% (Q1 = 77; Q3 = 90 %) at 24 h (Wilcoxon test corrected *p* = 0.002), and increased to +181% (Q1 = 163; Q3 = 198) at 12 weeks (*p* = 0.004). In parallel, water diffusion increases in the lesion (hypointense regions) (Figure 5.2).

**FIGURE 5:**
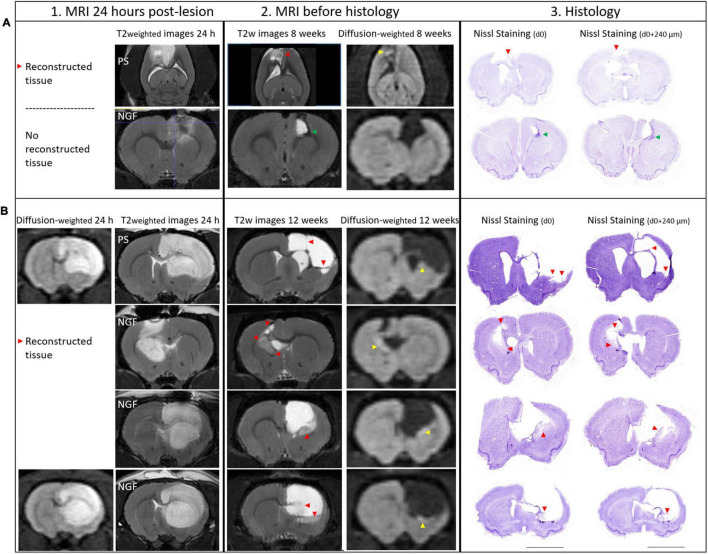
MRI at 24 h (vertical section 1), 8 and 12 weeks (section 2) and Histology of brain sections (section 3) and characterization of the lesion: the reconstructed tissue at 8 and 12 weeks post-injury. **1.** At 24 h post-lesion, T2 weighted and diffusion weighted images show hyperintense edematous regions, vasogenic and cytotoxic edema, respectively. Diffusion images were not acquired for all rats at 24 h. Lesions are characterized by an increase in T2 signal and an decrease in water diffusion (hyperintense area on diffusion-weighted images). **2.** As tissue becomes necrotic, T2 and water diffusion increase. The reconstructed tissue was observed both on MRI slices and at the exact same location on histology sections (red arrowheads). Restricted water diffusion is evidenced by hyperintensities in the core of the reconstructed tissue (yellow arrowheads). **3.** Nissl staining on two coronal sections separated by 240 μm and the corresponding MRI slice (section 2, *thickness 300 μm*) showing the reconstructed tissue. **(A)** Brain images from two rats sacrificed at 8 weeks are shown: PS rat (upper panel) and NGF rat (lower panel). Axial images are shown for the PS rat because a MRI artifact, probably coming from a blood clot, caused a distortion in the coronal slices near the skull. Reconstructed tissue is detectable in PS rat, while it is not in NGF rat, because the ventricle is dilated and reaches the skull. In the perilesional area of NGF rat, a hypointense area on T2 images appears (green arrowhead), compatible with newly generated cells (DCX+ and BrdU+ cells, data not shown). **(B)** Brain images from four different rats that were sacrificed at 12 weeks post-injury are shown. The upper panel shows PS group, while the lower three panels the NGF group. For the PS rat, thin filaments of reconstructed tissue could be detected by MRI (red arrowheads). Diffusion weighted images corresponding to the same section are shown on the right. Histological assay may damage the fragile neotissue (Nissl staining). Scale bars: 5 mm. MRI: magnetic resonance imaging; PS: physiological saline; NGF: Nerve Growth Factor; DCX: doublecortin and BrdU: BrdU: 5′-bromo-2-deoxyuridine.

Interestingly, at 12 weeks post-insult, instead of an increase in T2 signal, we observed a decrease in some regions. For the first time, we observed less T2 hyperintense regions of interest within the lesion (Figure 5.2, red arrowheads). These regions of interest (ROIs) corresponded strictly to neo-tissue within the initial lesion visualized on histological sections (Figure 5.2, red arrowheads) even when this neo-tissue formed very thin bands (see below, section *reconstructed tissue*). These ROIs were not observed at 24 h post-lesion but could be seen at an intermediate time point we investigated as a pilot test in 4 animals sacrificed at 8 weeks post-insult (Figure 5.2.A). However, they did not occur in an animal presenting a dilated ventricle extending to the skull (Figure 5.2.A). At 12 weeks, T2 signal intensity was significantly smaller in these ROIs compared to the lesion, –38% (Q1 = –44; Q3 = –32 %) (Wilcoxon test corrected, *p* = 0.01), and higher than the contralateral normal tissue, +82% (Q1 = 77; Q3 = 98 %), (Wilcoxon test corrected, *p* = 0.01). Moreover, water diffusion was isotropic and significantly reduced in the core of these ROIs compared to normal tissue (Trace_TR_ = 595 ± 84 μm^2^/s vs. 745 + 43 μm^2^/s; *p* = 0.001). Reduced diffusion appears hyperintense in Figure 5.2 (right, yellow arrowheads) on diffusion-weighted images while it appears hypointense on calculated and quantitative images of average water diffusion diffusivity (trace) (data not shown; see calculated values). Thus, water diffusion was restricted in the core of the neo-tissue. NGF treatment had no effect on theses MRI parameters. Importantly, diffusion and T2 MRI provided biomarkers for reconstructed tissues within a chronic lesion.

### Long-Term Nerve Growth Factor Treatment Promotes Tissue Reconstruction and Remodeling

Histological analyses (immunohistochemistry and immunofluorescence) were performed to characterize inflammation and glial scarring at the lesion site, proliferation, neurogenesis (progenitors, immature and mature neurons) and angiogenesis (neovascularization) *in the reconstructed tissue*, in NGF- and PS-treated rats.

#### Glial Scar Characterization

**FIGURE 6:**
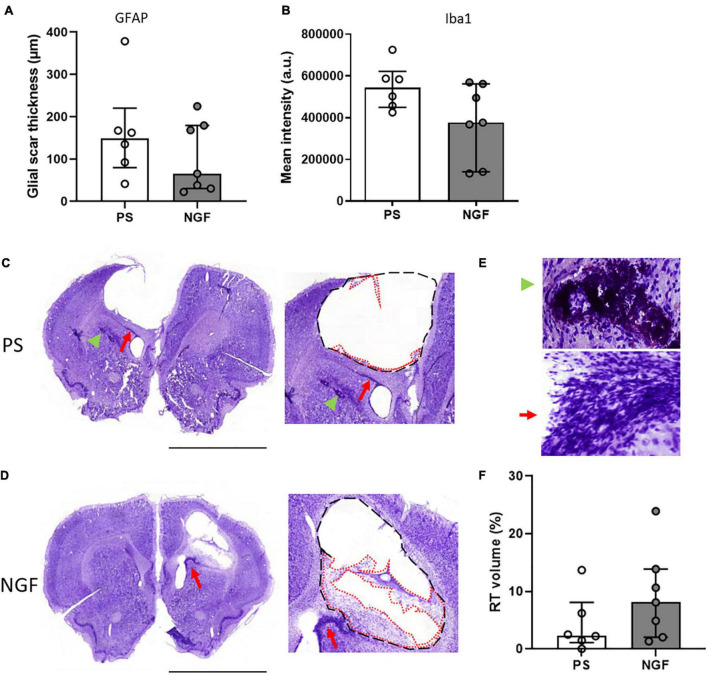
Histology of brain sections and characterization of the lesion: the glial scar and the reconstructed tissue at 12 weeks post-injury. **(A)** Quantification of the glial scar thickness (expressed in μm) based on GFAP mean intensity (fluorescence) in PS and NGF groups. There was no significant difference between the groups. **(B)** Quantification showing the microglia (Iba1 mean intensity) in the glial scar in PS and NGF groups. There was no significant difference between the groups. **(C,D)** Nissl staining on coronal sections (*left*) and magnifications (*middle*) showing the edges of the lesion (black dashed lines) and the reconstructed tissue (red dotted lines) 12 weeks post-injury in PS **(C)** and NGF **(D)** groups. A migration pathway is observed between the ventricle and the lesion edge [Panel **(C,D)**, Red arrow]. **(E)** magnifications showing pyknotic tissue (green arrowhead) and migration pathway (red arrow). **(F):** Graph showing the quantification of the reconstructed tissue (RT) normalized by the lesion volume in PS and NGF groups. All the graphs show the distribution of individual values and the median and the interquartile range. PS group: *n* = 6; NGF group: *n* = 7. Scale bars: 5 mm. PS: physiological saline; NGF: nerve growth factor; GFAP: glial fibrillary acidic protein; a.u: arbitrary unit.

The thickness of the glial scar at the edge of the lesion was measured 12 weeks post-injury in brain sections labeled with GFAP, an astrocytic marker and major component of the scar tissue. However, this tight tissue was not observed all around the lesion. There was no significant difference between the two groups of rats (*p* = 0.60) (Figure 6A). Together with astrocytes, microglia also migrated into the glial scar. We characterized these cells by Iba1 expression and found a similar expression in the two groups of rats (*p* = 0.07) (Figure 6B). Thus, NGF treatment did not show significant effects on glial scar remodeling.

#### Reconstructed Tissue

Nissl staining of brain sections revealed reconstructed tissue in rats sacrificed at 8 and 12 weeks, regardless of treatment group (Figures 5.3A,B). At 12 weeks post-injury, rats receiving NGF treatment tended to have slightly more reconstructed tissue, identified at the edge of the injury (median = 8.12%; Q1 = 3.5%; Q3 = 12.1%, Figure 6D) compared to the PS group (median = 2.39%; Q1 = 1.7; Q3 = 5.3%, Figure 6C) (*p* = 0.29, not significant; Figure 6F). Importantly, reconstructed tissue could be identified by histology and by T2 and diffusion MRI (see above). Migration pathways from the subventricular zone (SVZ) were seen in some animals of both groups at 8 and 12 weeks (Figure 5A, green arrowhead, Figures 6C–E, red arrows). Perilesional secondary degeneration and pyknotic nuclei (Zille et al., 2012) were seen in three rats out of eight in both groups and did not seem to be affected by NGF (Figures 6C,E, green arrowheads).

**FIGURE 7:**
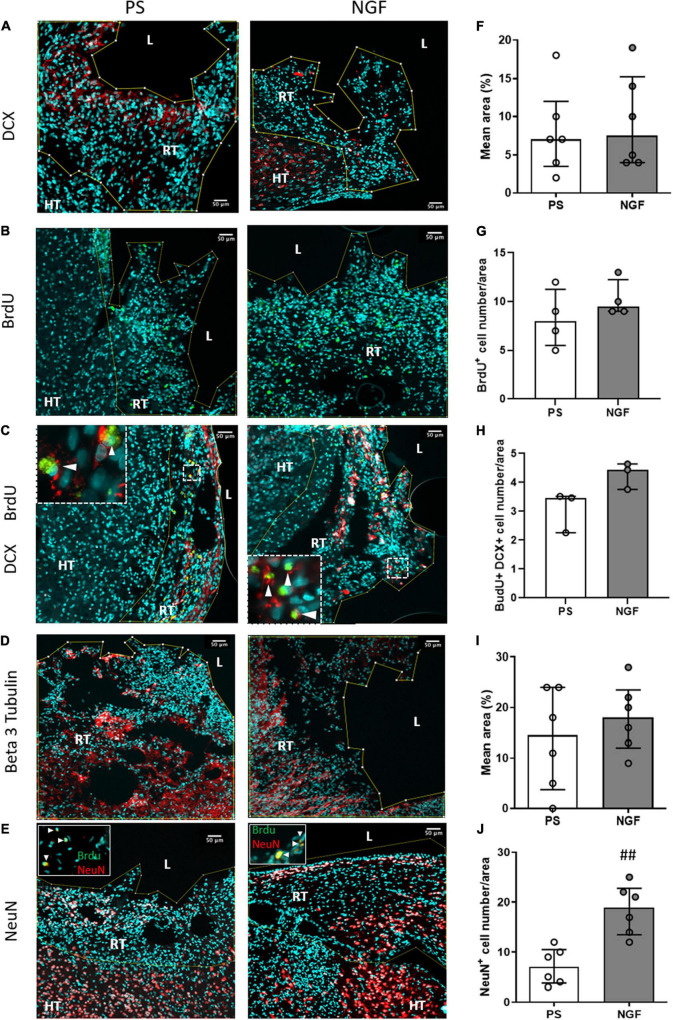
Histology of brain sections and characterization of the reconstructed tissue neurogenesis. Representative images and quantification of DCX **(A,F)**, beta 3 tubulin **(D,I)** mean intensity, NeuN **(E,J)** cell number (percentage of DAPI+ cells) and BrdU **(B,H)** cell number (percentage of DAPI+ cells) in the reconstructed tissue in PS (*left*) and NGF (*middle*) groups. Double staining with BrdU/DCX and BrdU/NeuN is shown in panel **(C,E)** (inserts). **(C)** Representative image showing BrdU (green) and DCX (red) staining. The inserts show the cells expressing both markers (white arrowheads). **(E)** Representative image showing NeuN (red) staining. The inserts show nuclei positives for BrdU (green) and NeuN markers (white arrowheads). No significant difference was observed for DCX, beta 3 tubulin and BrdU between the groups, as reported in the graphs **(F–I)**. A significant difference in percentage of NeuN positive-cells was observed between the groups (## *p* = 0.0043). All the graphs show the distribution of individual values and the median and the interquartile range. PS group: *n* = 6; NGF group: *n* = 7 except for BrdU. Scale bars: 50 μm. PS: physiological saline; NGF: nerve growth factor; DCX: doublecortin; NeuN: neuronal nuclei; BrdU: 5-bromo-2-deoxyuridine. L: lesion; RT: reconstructed tissue; HT: healthy tissue.

Immunofluorescence staining was performed to identify: cell proliferation using BrdU, neurogenesis using DCX for neuronal progenitors, beta 3 tubulin for immature neurons and NeuN for mature neurons; gliogenesis using GFAP for astrocytes and Iba1 for the microglia; and angiogenesis using PDGFRB to identify pericytes and new vessels. We did not find difference in astrocyte (Figure 3D), microglia (Figure 3E) and pericyte (Figure 3F) expression in NGF compared to PS group (Mann-Whitney test; GFAP: *p* = 0.93; Iba1: *p* = 0.62; PDGFRB: *p* = 0.80). Interestingly, neuronal markers were found in several clusters within the reconstructed tissue. Expression of these neuronal markers in the reconstructed tissue was different in the two groups of rats. Quantification shows a significant increase in the percentage of mature neurons (NeuN-positive) in the reconstructed tissue of the NGF compared to PS rats (*p* = 0.0043) (Figures 7E,J). As for the other neural markers, the percentage of positive cells was similar in the two groups of rats (Mann-Whitney test; DCX: *p* = 0.73; beta 3 tubulin: *p* = 0.55) (Figures 7A,F and Figures 7D,I, respectively). BrdU labeling (Figures 7B,G) was observed in areas where DCX+ cells were found (Figure 7C) and was rare in the perilesional tissue. We did not find an effect of NGF on BrdU labeling (Mann-Whitney test *p* = 0.31 with *n* = 4 per group; Figure 7G). A double staining was carried out in two animals per group and less than 2% of the total cells found were BrdU+/NeuN+ (PS: 0.09% and 0.28%, NGF: 0.12% and 1.95% cells) (inserts Figure 7E). Most of NeuN+ cells were BrdU-. DCX+ cells were also found near the ventricle, in the perilesional tissue (Figure 7A) and beta 3 tubulin+ cells were also observed in the perilesional tissue. However, many cell clusters of reconstructed tissue sometimes far from the ventricle were characterized by a high concentration of BrdU+/DCX+ cells whereas the perilesional tissue contained few of these cells (Figure 7C). Near the ipsilesional ventricle, these clusters contained less DCX+ cells than the perilesional tissue (Figure 7A, NGF rat). Both observations support our interpretation of a *de novo* tissue. Although the number of BrdU+/DCX+ cells increased in NGF compared to PS-treated rats, the Mann-Whitney test was not significant, likely because of the small sample (4.4% versus 3.4%; PS: *n* = 3; NGF *n* = 3; Mann-Whitney test; *p* = 0.10; Figure 7H).

**FIGURE 8:**
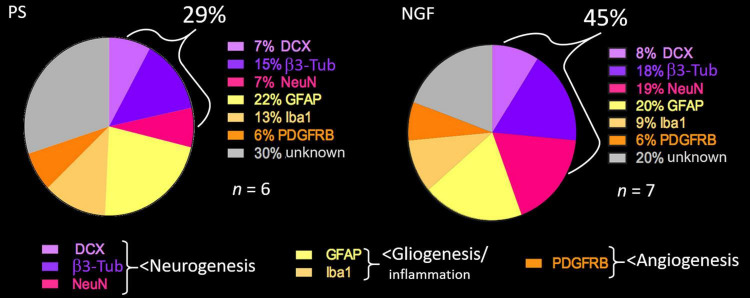
Pie chart showing the percent of cell types detected in the brain sections of PS (*left*) and NGF (*right*) groups identified by histology 12 weeks post-injury. PS group: *n* = 6; NGF group: *n* = 7. PS: physiological saline; NGF: nerve growth factor; DCX: doublecortin; NeuN: neuronal nuclei; GFAP: glial fibrillary acidic protein; PDGFRB: platelet-derived growth factor receptor beta; β3-Tub: beta 3 tubulin.

The percent of the different cell populations identified in our study is reported in the pie-chart in Figure 8. NGF treatment favors neuronal differentiation and survival over glio- and angiogenesis.

### Nerve Growth Factor Effect on Behavior Tests

There was no effect of a 10-week NGF treatment on rat weight. Chronic treatment with NGF did not produce any sign of stress, nasal irritation, nor did it cause death. Two rats showed unilateral vibrissae immobilization which disappeared on cessation of treatment (2 days) and did not reappear when treatment resumed. The rats showed normal behavior throughout the study.

#### Grip Strength Test

**FIGURE 9:**
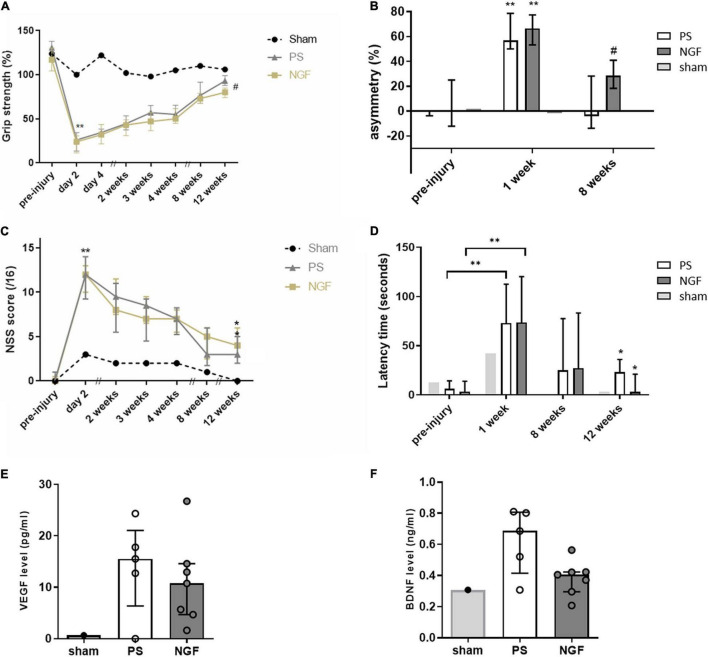
Behavioral tests following injury to the sensorimotor cortex. **(A)** Grip strength test shows the grip strength of the front paw contralateral to the injected hemisphere compared to the other paw, expressed as %. The Wilcoxon test compares the values measured 2 days post- compared to the pre-injury ones for the PS (gray triangles) and NGF (light yellow squares) groups (**p < 0.01). The Bonferroni corrected Mann Whitney test compares the results between the PS and the NGF groups, significant at 12 weeks post-injury (^#^*p* = 0.014). **(B)** The limb-use asymmetry test measures the asymmetric limb use, meaning the difference of use for the ipsilateral - contralateral paws compared to the number of supports in %. Both groups were significantly impaired on the contralateral limb 1 week post-injury compared to pre-injury values (Wilcoxon test, **p < 0.01). The difference between PS (white bars) and NGF (dark gray bars) groups is significant 8 weeks post-injury (Mann Whitney test, #*p* < 0.05). **(C)** The NSS shows the sensorimotor deficits after malonate injection (scored out of 16). Both groups were significantly impaired 2 days post-injury compared to pre-injury values (Wilcoxon test, **p < 0.01). A significant improvement in NSS was observed in both groups [PS (gray triangles) and NGF (light yellow squares)] 12 weeks compared to 2 days post-injury (*p < 0.05). **(D)** The anxiety test showing that the latency to take out two front limbs was significantly increased between the pre-injury and 1 week post-injury values for both groups [PS (white bars) and NGF (dark gray bars)] (Wilcoxon test, **p < 0.01). The latency significantly decreased between 1 week and 12 weeks post-injury in both groups, independently of the treatment (*p < 0.05). The Mann-Whitney test did not show any difference between the two groups 12 weeks post-injury. The graphs show the median values and their interquartile ranges; For **(A–D)**, sham: *n* = 1; PS group: *n* = 9; NGF group *n* = 9; *n* corresponds to the number of individuals. **(E,F)** Notch box plots showing the level of VEGF (*left*) and BDNF (*right*) measured in the plasma of PS and NGF groups 12 weeks post-injury. The graphs show the distribution of individual values and the median and the interquartile range. PS group: *n* = 5; NGF group *n* = 7. PS: physiological saline; NGF: nerve growth factor; NSS: neurological severity scale; VEGF: Vascular endothelial growth factor; BDNF: brain-derived growth factor.

The grip test was used to identify the dominant paw, which was the one targeted by the injury (contralateral hemisphere), and to evaluate the strength of the two forepaws after injury and treatment. The percent of the strength of the dominant paw in PS- and NGF-treated rats is shown in Figure 9A. Before the injury, the dominant paw reached, on average, 21% greater force than the non-dominant one. After the injury, as well after NGF or PS administration, the strength of the unaffected (non-dominant) paw was not modified. At day 2 post-injury, the deficit in the dominant paw was clear and measurable in all injured rats compared to pre-injury values (Wilcoxon test, *p* < 0.01). A difference between the NGF and PS-treated groups was observed only at 12 weeks post-injury. Both groups displayed the same recovery rate except at 12 weeks. In detail, the PS group showed a progressive recovery reaching 93% (Q1 = 88%, Q3 = 99%) of strength compared to 131% pre-injury, thus still significantly different (Wilcoxon test, *p* = 0.015). The NGF group achieved 80% (Q1 = 74%, Q3 = 85%) of strength compared to 117% pre-injury. This indicates a significant difference compared to pre-injury values (Wilcoxon test, *p* = 0.015), as well as a significantly poorer recovery following NGF treatment compared to PS (corrected p for multiple comparisons = 0.024, Figure 9A).

#### Limb-Use Asymmetry Test

This test is used to assess the spontaneous use of the front limbs of rodents. The percent of asymmetry (Figure 9B) makes it possible to measure the motor deficits of the entire limb (shoulder, elbow, paw). All rats supported their body weight during rearing symmetrically using the two forelimbs before injury. Post-injury, asymmetry was greatly increased after 1 week, indicating a significant preference for use of the ipsilateral paw. The deficit recovered 8 weeks post-injury (PS: *p* = 0.0078 and NGF: *p* = 0.0039, Wilcoxon test). Importantly, the percent of asymmetry was significantly lower for the PS compared to the NGF group 8 weeks post-injury (*p* = 0.020 Mann-Whitney test Bonferroni corrected).

#### Neurological Severity Scale

The evaluation of the sensorimotor deficit using the NSS revealed a significant increase (*p* < 0.01) in the total score at day 2 post-injury in all the rats, highlighting the consequences of the injury targeting the sensorimotor cortex (Figure 9C). Rats in NGF- and PS-treated groups recovered from sensorimotor deficits 12 weeks post-injury (*p* < 0.05). There was no statistically significant difference in the recovery rate between the two groups (*p* = 0.14).

#### Anxiety Test

The consequences of motor cortex injury on rat anxiety and its recovery were evaluated by the emergence test. The latency to exit the dark compartment was significantly increased 1 week post-injury in all rats (*p* < 0.0001, Figure 9D). Since NGF is known to act on stress modulation (Aloe et al., 2012), this effect was monitored. There was no particular effect of the NGF treatment on the latency time, however, this was significantly reduced 12 weeks post-injury in both groups, compared to the respective baseline values (*p* < 0.05, Wilcoxon test).

### Circulating Growth Factors

The levels of VEGF and BDNF were measured in the plasma collected 12 weeks post-injury. The plasmatic concentration of both factors was higher in injured compared with the non-injured sham rat (Figures 9E,F). However, VEGF and BDNF levels were not significantly different between PS and NGF groups, although a noticeable decrease of BDNF was observed in the NGF group (*p* = 0.07, Mann-Whitney test).

## Discussion

The primary need for ischemic stroke therapy is to find a valid treatment inducing functional recovery associated with brain tissue reconstruction. In this preclinical study, we show that intranasal NGF treatment promotes tissue regeneration with a significantly higher proportion of neurons observed twelve weeks after brain injury. A ten-week NGF treatment showed no adverse events although functional recovery was slightly affected. The design of the study was critical to identify the ideal duration of a long treatment, as well the optimal dose of NGF, which remains a challenge. More importantly, diffusion and T2 MRI provided for the first time biomarkers of cerebral reconstruction.

Nerve growth factor (NGF) has been used with hope in clinical trials, since some benefits have been reported in Alzheimer (Jönhagen et al., 1998) or Traumatic Brain Injury (TBI) patients (Chiaretti et al., 2008, 2017). However, important concerns limit its use. Some studies report undesirable side-effects, consequent to intramuscular (Costa et al., 2020), intravenous (Petty et al., 1994), intracerebroventricular infusion (Jönhagen et al., 1998), and subcutaneous injection in neuropathic (diabetic) patients (Apfel, 2002), or failed to demonstrate significant beneficial effects (Apfel et al., 2000). The lack of efficacy may be explained by the low doses (0.1 μg/kg) used in neuropathic patients (Apfel, 2002), for example. To avoid its pro-nociceptive activity, NGF has been administered intra-parenchymally into the brain by neurosurgery *via* cell or gene therapy approaches (Tuszynski et al., 2005, 2015). A recent meta-analysis of randomized controlled trials testing intramuscular injection of NGF over ten years in China concluded positively about its safety and efficacy for the treatment of neurological diseases (Zhao et al., 2015). However, most of the cited trials were not blinded. Moreover, NGF intramuscular injection is still a subject of active research, notably for use as a model of hyperalgesia (Costa et al., 2020). What emerges so far is that NGF therapy requires further investigations to find the best combination of administration route and dosage, to avoid negative side effects.

This study aims to respond, in part, to these needs by testing intranasal delivery and a dose of NGF that is intermediate, compared to the ones reported in the literature. Intranasal delivery of neurotrophins, such as NGF, to the brain *via* the olfactory bulb and the trigeminal nerve pathways is well tolerated (Alcalá-Barraza et al., 2010) and leads to substantial concentrations of neurotrophic products (Aloe et al., 2012), in particular in the frontal and parietal cortices (Lioutas et al., 2015), which are most often affected by stroke. Intranasal NGF can also pass into the cerebrospinal fluid (Lochhead and Thorne, 2012) and can directly target the lesion. In our model, the lesion core can have direct contact with the cerebrospinal fluid and also the lateral ventricle lumen and thus the SVZ. This fact could be relevant for explaining the origin of the neo-tissue in the cavity and the effect of NGF. Peripheral delivery through absorption (1) into olfactory blood vessels and entry into the general circulation, and (2) into olfactory lymphatic vessels draining to the deep cervical lymph nodes of the neck is reportedly minimal (Alcalá-Barraza et al., 2010; Lochhead and Thorne, 2012). Moreover, NGF does not need to be encapsulated as do other growth factors such as transforming growth factor-alpha (Guerra-Crespo et al., 2010). Finally, intranasal administration of NGF seems a good strategy to avoid its pro-nociceptive effects observed in intramuscular injection (Chiaretti et al., 2017; de Bellis et al., 2018).

Very few studies have tested intranasal NGF treatment over a prolonged period of time after an acute brain lesion. In humans, the longest NGF treatment (40 days, in 4 periods of 10 days) is described in a child after TBI, and was demonstrated to be safe (Chiaretti et al., 2017). As for ischemic stroke, the longest intranasal NGF treatment was six days in rats (post MCAo – middle cerebral artery occlusion -) (Li et al., 2018). A prolonged treatment may be needed to improve recovery, as described for transforming growth factor-alpha (Guerra-Crespo et al., 2010). This molecule, given in one dose per week for 4 weeks, induced neural progenitor proliferation and migration and enhanced impaired forelimb use in rats (Guerra-Crespo et al., 2010). Therefore, we chose to perform the NGF treatment over ten weeks.

Together with treatment duration, the choice of NGF dose is pivotal to find the right balance between efficacy and absence of side effects. Experimental studies in brain injuries have used high doses (60 μg/day/rat post-MCAo for 6 days) (Zhu et al., 2011) or lower doses (5 μg/day/rat for 7 days post-TBI) (Young et al., 2015), the latter being ineffective. In humans, the high dose of 0.2 mg/kg/day was used in a case report of TBI, cited above (Chiaretti et al., 2017). Based on this evidence and on the duration chosen for the treatment, we decided to use an intermediate dose of NGF (5 μg/kg/day), which was previously described as safe for a 7 day treatment (Young et al., 2015).

For the design of the current study, we thoroughly examined the data in the literature to decide how to target tissue reconstruction and functional recovery in our model of brain lesion in rats. We considered that, given the length of the early subacute phase, ranging from 7 days to 3 months post-stroke (Bernhardt et al., 2017), and the complexity of cellular events, the subacute phase does not represent a steady state. Thus, we hypothesized that a therapeutic protocol with two interventions (five weeks each) and a wash-out period in between (two weeks) is suitable to test to assess the effect of a NGF treatment on each period. Daily observation and behavior tests showed that during the first five week period, no adverse effects were observed in rats. We used validated tests that are sensitive and robust to evaluate a possible effect due to the NGF treatments (Vaysse et al., 2015b; Davoust et al., 2017). Although we observed a delay in motor recovery consequent to the second five-week period of treatment, no side effects were evident. Our interpretation is that it might be worthwhile to decrease the doses of NGF or space them out, to avoid the accumulation of the neurotrophin and decrease detrimental effects while keeping the benefit on neurogenesis. This hypothesis warrants further testing in a future experimental study.

In the peripheral nervous system, NGF exerts trophic effects primarily on sympathetic neurons and small diameter sensory neurons that mediate pain and temperature sensation (Apfel, 2002). The late-onset negative effect of NGF on grip strength and upper limb elevation reported in the present study might suggest an effect on peripheral nerves or muscles, thus not excluding the occurrence of peripheral side effects. This could be due to the overstimulation of the sensory fibers and increased sensitivity to pain. However, we did not expose the animals to painful stimuli to test this hypothesis. Furthermore, NGF treatment did not affect the anxiety levels. Animals did not present signs of suffering such as poor grooming or weight loss. As stated above, the distribution of NGF in the periphery is minimal through intranasal delivery, therefore hyperalgesia seems rather unlikely. Many interpretations are reasonable, based on the effects that NGF has on cell activation, proliferation, differentiation, migration, survival and apoptosis (Cheng et al., 2009a; Aloe et al., 2015). In addition, this molecule has a physiological role on angiogenesis, neuronal development, wound healing, immune regulation of the hypothalamic-pituitary-adrenal axis and stress activity (Cheng et al., 2009a; Aloe et al., 2012). In our study, we found an important decrease of BDNF at twelve-weeks after NGF treatment, indicating that exogenous NGF might have negatively modulated endogenous BDNF signaling and impacted neuronal function and recovery (Nijs et al., 2015). Moreover, we cannot exclude that inhibitory connections were formed between the perilesional and neo-formed tissue that would slightly impair limb recovery.

As stated earlier, the different effect of NGF on functional recovery during the two periods of treatment could be explained by the physiological events associated with tissue injury and reconstruction. Neurogenesis is high during the first month after ischemic stroke and decreases thereafter (Arvidsson et al., 2002; Jin et al., 2006; Kuge et al., 2009). Although low, persistent neurogenesis has been described until four months after stroke (Thored et al., 2006). Our results suggest that the dose of NGF needed to promote neurogenesis, to sustain differentiation and survival should be adapted to these physiological events, being low during the first two months and high during the third month after brain lesion. There are five distinct time phases after stroke: hyperacute, acute, early subacute, late subacute and chronic (Joy and Carmichael, 2021). We suggest that there might be one more, a mid-subacute phase between two- and three months after stroke where plasticity reaches a plateau (see Figure 2). In this scenario, pharmacological intervention would find fewer cellular targets. Overall, the importance of treatment duration is evidenced in clinical studies, where for example a three month administration of fluoxetine improved functional recovery (Chollet et al., 2011), whereas a six-month therapy was not effective (Hankey et al., 2020).

Previous studies on the intranasal strategy in ischemic stroke evaluated the rate of neural progenitor proliferation (Cheng et al., 2009b; Guerra-Crespo et al., 2010; Zhu et al., 2011). Only a few experimental studies characterized and quantified the volume of reconstructed brain tissue after injury. Arvidsson et al. (2002) have described that only 0.2% of neurons are replaced by spontaneous neurogenesis six weeks after stroke, since most of new mature neurons do not survive. However, eight weeks after, we could detect substantial tissue reconstruction and twelve weeks after, we quantified 2.4% of reconstructed tissue in control rats, which is the first evidence quantifying a spontaneous regeneration after a brain lesion. This information highlights the importance of long-term follow-up. In addition, we show for the first time that a non-invasive treatment can nearly quadruple this value (8.1%) and lead to a substantial tissue reconstruction within the lesion (∼8.5 mm^3^). Interestingly, the amount of reconstructed tissue we report here is higher even than that observed (< 0.56 mm^3^) twelve weeks after an intracerebral graft of stem cells (Buhnemann et al., 2006). High rates of reconstruction have been described with invasive approaches including stem cell graft or biomaterials (Park et al., 2002; Kim et al., 2011; Bible et al., 2012; Davoust et al., 2017; Ghuman et al., 2018).

The proportion of mature neurons (NeuN-positive) found in some clusters of the reconstructed tissue of the NGF group was 19%, which reflects the physiological amount in the parenchymal tissue, versus 7% in the PS group. We assessed late neurogenesis with BrdU given 40 days post-injury. Interestingly, some BrdU+/NeuN+ neurons were found. Since migration pathways from the SVZ to the peri-infarct area were observed in both groups of rats, it seems that mature neurons could arise from SVZ stem cell differentiation after 40 days, though in too low amounts to fully reconstruct a large lesion. BrdU-negative NeuN-positive neurons could have been formed during early post-injury neurogenesis from SVZ stem/progenitor cells. As for neural progenitors and immature neurons, we observed 8% and 18%, respectively, after NGF administration, compared to 7% and 15% in PS rats. Thus, their number seems only slightly affected by NGF treatment. There was a trend toward more BrdU+/DCX+ cells after NGF treatment. This indicates that a neuronal regeneration is still possible three months post-injury. This is in agreement with previous studies (Cheng et al., 2009b; Zhu et al., 2011) showing that NGF treatment failed to increase cell proliferation during the early phase (1d or 28d after MCAo in mice) but improved survival of newly generated neuronal cells. Moreover, our results suggest that most of the mature neurons found might either originate from early endogenous SVZ-associated neurogenesis or could originate from reactive astrocytes. Reactive astrocytes may give rise to neurons by dedifferentiation then neuronal transdifferentiation, or via transit-amplifying cells, which are transformed by de-repression of a latent neurogenic transcriptional program (Shimada et al., 2012; Magnusson et al., 2020). Indeed, the latter mechanism has been evidenced in brain-injury models in rodents and the amounts of cells generated seem effective for cell replacement strategies (Magnusson et al., 2014, 2020). Interestingly, Scardigli et al. (2014) demonstrated that NGF is crucial for SVZ-derived neurogenesis and for proliferation and differentiation of astrocytes. Moreover, NGF rescued defects in astrocytes of NGF-neutralized mice *in vitro* and intranasal NGF rescued proliferation of SVZ progenitors (Scardigli et al., 2014). The percent of astrocytes in the reconstructed tissue was similar in the two groups of rats (20% NGF *vs*. 22% PS). This data, compared with the one on mature neurons (19% NGF *vs.* 7% PS) indicates a ratio strikingly in favor of neurons after NGF treatment. Altogether, the data on vascularization (PDGFR+), gliogenesis and inflammation (GFAP+ and Iba1+) indicates that NGF increased neurogenesis and cell survival, confirming the literature (Zhu et al., 2011). Newborn neurons from the SVZ that migrate into the peri-infarct cortex have been shown to be functional and critical for post-injury recovery (Liang et al., 2019). This perilesional plasticity might lead to the formation of connections with new neurons from the reconstructed tissue, potentially allowing them to integrate in a neural network useful for functional recovery (Modo, 2019).

Finally, we observed 12 weeks after the lesion that non-invasive diffusion and T2 MRI could irrefutably highlight the neo-tissue being reconstructed. The new tissue was characterized by an intermediate T2 value between normal and damaged tissue. This suggests an intermediate water content or an intermediate water mobility. This would be consistent with loose tissue being reconstructed within a lesion. Second, this tissue exhibited a core where diffusion was restricted compared to normal tissue in accordance with a dense extracellular matrix. To our knowledge, these biomarkers have never been proposed in the literature.

We acknowledge several limitations in this study. We made the proof of principle that newly regenerated tissue within brain injury can be remodeled by a growth factor toward neurogenesis. However, we have not fully established the origin of newly formed tissue and have not proven that it has an impact on functional recovery. A longer time might be needed for a neotissue to become functional. Also, a lesion resulting in large cavity is not comparable with most ischemic stroke lesion in humans, where fibrosis is higher, which has a bearing on ability to study plasticity and functional recovery. The higher white over gray matter ratio and the amount of myelin in human brain might influence the formation of fibrosis. Furthermore, aberrant connections between the neo-tissue and the peri-infarct areas can occur and induce maladaptative plasticity such as epilepsy, pain or inhibitory connections that could limit recovery. Although we did not observe any adverse events like epilepsy or pain, we cannot exclude that inhibitory connections were formed. A reconstructive plasticity must be paired with a sustained rehabilitation to insure correct rewiring of newly generated neurons. The high amount of behavioral testing and social interaction (handling for NGF treatment) in this study (adding up to 1.5 h a week for each rat), could be to some extent compared to intense rehabilitation.

In conclusion, the design of an optimal long-term therapy needs some refinement of the dose and the window of treatment after stroke. Nevertheless, we demonstrated that the nose-to-brain pathway is a valid strategy for repeated and non-invasive administrations of NGF treatment. More importantly, NGF treatment enhanced cerebral reconstruction and neuronal survival which could be evidenced non-invasively by *in vivo* diffusion and T2 MRI.

## Data Availability Statement

The original contributions presented in this study are included in the article, further inquiries can be directed to the corresponding author.

## Ethics Statement

The animal study was reviewed and approved by Comité d’éthique pour l’expérimentation animale Midi-Pyrénées (protocol n° 16780).

## Author Contributions

NC: experimental work, analysis and interpretation of the data, data curation, and draft and editing of the manuscript. FD: MRI expert, experimental work, and analysis and interpretation of the data. AL: experimental work, ImageJ macro creation for glial scar thickness, and editing of the manuscript. LR: experimental work. AM: bibliographic work. CC: study design, experimental work, analysis and interpretation of the data, draft and editing of the manuscript, and funding acquisition. IL: study conception and design, analysis and interpretation of the data, draft and editing of the manuscript, funding acquisition, and project administration. All authors contributed to the article and approved the submitted version.

## Conflict of Interest

The authors declare that the research was conducted in the absence of any commercial or financial relationships that could be construed as a potential conflict of interest.

## Publisher’s Note

All claims expressed in this article are solely those of the authors and do not necessarily represent those of their affiliated organizations, or those of the publisher, the editors and the reviewers. Any product that may be evaluated in this article, or claim that may be made by its manufacturer, is not guaranteed or endorsed by the publisher.
